# Associations of Metals with Cognitive Function and Blood-Based AD Biomarkers in Midlife

**DOI:** 10.1093/geroni/igaf122.2215

**Published:** 2025-12-31

**Authors:** Sithara Vivek, Shannon Sullivan, Jesse Seegmiller, Eric Grodsky, Chandra Muller, John Warren, Bharat Thyagarajan

**Affiliations:** University of Minnesota, Minneapolis, Minnesota, United States; University of Minnesota, Minneapolis, Minnesota, United States; University of Minnesota, Minneapolis, Minnesota, United States; University of Wisconsin–Madison, Madison, Wisconsin, United States; The University of Texas at Austin, Austin, Texas, United States; University of Minnesota, Minneapolis, Minnesota, United States; University of Minnesota, Minneapolis, Minnesota, United States

## Abstract

Dysregulation of essential metal homeostasis and metalloproteinase activity contribute to AD pathology. While previous studies have examined metal concentrations and AD biomarkers in CSF, their associations in blood remain largely unexplored. In this study, we investigated the association between circulating metals and blood-based biomarkers of AD and cognitive function in the High School and Beyond (HS&B:80/21) study (n = 4,150, mean age= 58 ± 1 years). We analyzed whole-blood levels of copper, manganese, selenium, zinc, arsenic, and lead (measured using inductively coupled plasma mass spectrometry) with composite cognitive function score and blood-based AD biomarkers (p-Tau 181, NfL, GFAP) measured using Quanterix Simoa system. Metals and AD biomarkers were log-transformed and standardized. Linear regression models were adjusted for age, sex, race, smoking status, education, BMI, eGFR and APOE e4 allele status. Benjamini-Hochberg correction was used for multiple testing. Several essential metals were positively associated with cognitive function, including selenium (β= .062, p < 0.0001, manganese (β= .051, p < 0.0001), and zinc (β= .025, p= .03), while copper β= -.107, p < 0.0001) was inversely associated. Additionally, several essential metals were inversely associated with AD biomarkers. Higher selenium and copper levels were associated with lower p-Tau 181 (Se: (β= -.043, p < 0.0001; Cu: (β= -.116, p < 0.0001). Selenium was also inversely associated with NfL (β = -.036, p = 0.03), while zinc showed a marginal inverse association with GFAP (β = -.035, p = 0.05). Our findings highlight the complex role of metals in AD and suggest that essential metals, particularly Selenium, may protect against cognitive decline and neurodegeneration.

